# Salivary levels of eluents during Invisalign™ treatment with attachments: an in vivo investigation

**DOI:** 10.1186/s40510-024-00522-6

**Published:** 2024-06-03

**Authors:** Larissa Stocker, Sevasti-Kiriaki Zervou, Spyridon N. Papageorgiou, Stephania Karakousoglou, Theodoros Triantis, Anastasia Hiskia, George Eliades, Theodore Eliades

**Affiliations:** 1 Clinic of Orthodontics and Pediatric Dentistry, Center for Dental Medicine, University of Zurich, Plattenstrasse 11, Zurich, 8032 Switzerland; 2 Laboratory for Photo-Catalytic Processes and Environmental Chemistry, Institute of Nanoscience and Nanotechology, National Center for Scientific Research “Demokritos”, Agia Paraskevi, Greece; 3 Private practice, Athens, Greece; 4 Department of Biomaterials, School of Dentistry, National and Kapodistrian University of Athens, Athens, Greece

## Abstract

**Background:**

The aim of the present study was to investigate qualitatively and quantitatively the elution of substances from polyester-urethane (Invisalign™) aligners and resin composite attachments (Tetric EvoFlow) in vivo.

**Methods:**

Patients (*n* = 11) treated with the aligners and attachments (16 per patient, without other composite restorations) for an average of 20 months, who were planned for attachment removed were enrolled in the study. Patients were instructed to rinse with 50 mL of distilled water upon entry and the rinsing solution was collected (before removal). Then, the attachments were removed with low-speed tungsten carbide burs for adhesive residue removal, a thorough water rinsing was performed immediately after the grinding process to discard grinding particle residues, and subsequently, after a second water-rinsing the solution was collected for analysis (after removal). The rinsing solutions were analyzed for targeted (LC-MS/MS: Bis-GMA, DCDMA, UDMA, BPA) and untargeted (LC-HRMS: screening of leached species and their degradation products) compounds.

**Results:**

Targeted analysis revealed a significant reduction in BPA after attachment removal (4 times lower). Bis-GMA, DCDMA, UDMA were below the detection limit before removal but were all detectable after removal with Bis-GMA and UDMA at quantifiable levels. Untargeted analysis reviled the presence of mono-methacrylate transformation products of Bis-GMA (Bis-GMA-M1) and UDMA (UDMA-M1), UDMA without methacrylate moieties (UDMA-M2), and 4-(dimethylamino) benzoic acid (DMAB), the degradation product of the photo-initiator ethyl-4-(dimethylamino) benzoate (EDMAB), all after attachment removal. Several amino acids and endogenous metabolites were also found both before and after removal.

**Conclusions:**

Elevated levels of BPA were traced instantaneously in patients treated with Invisalign™ and flowable resin composite attachments for the testing period. BPA was reduced after attachment removal, but residual monomers and resin degradation products were found after removal. Alternative resin formulations and attachment materials may be utilized to reduce eluents.

## Introduction

Over the past decades, clear aligners have become an increasingly popular treatment modality for a wide spectrum of malocclusions. Although treatment with conventional fixed appliances may deliver superior treatment outcomes [1, 2], the increasing demand for a discreet treatment method and vigorous advertising by manufacturing companies continue to boost the demand for aligners not only among adult patients but also among teenagers [3]. Furthermore, aligners facilitate good oral hygiene [4], are less painful than conventional braces [5] and the overall treatment duration tends to be reduced [6, 7].

While aligner therapy was initially limited to correcting minor rotations and tipping deviations, the introduction of bonded resin attachments considerably expanded the spectrum of indications. The additional grip and range of attachment size and shape allow for control of tooth movement in all three planes of space, including buccolingual inclination changes and vertical repositioning [8]. Despite these notable clinical advantages, the use of composite attachments is not free of concern. The acid etching-mediated bonding annihilates enamel structure integrity [9, 10], increases the risk of decalcification and white spot lesions [11], and alters the enamel’s optical properties [12, 13]. Moreover, the potential release of cytotoxic and estrogenic monomers and compounds such as Bisphenol-A (BPA) could have hazardous health effects. In comparison to conventional bracket bonding where only the margins of the resin are exposed to the oral cavity, the exposed surface of attachments is estimated to be substantially larger with the number and shape of attachments used [8] varying the actual surface exposed, posing a higher risk of leaching potentially harmful factors.

BPA is one of the most common synthetic chemicals, primarily used in the production of polycarbonate plastics and epoxy resins, which have a large range of applications in food packaging, household utensils, toys, and the automobile and aerospace industry. Its release has become of paramount interest over the past decades among researchers and legislative bodies alike, as various negative health effects even after low levels of BPA exposure [14] have become evident. Due to its similar chemical structure to natural estradiol, it is capable of mimicking hormonal actions and disrupting the endocrine system. Therefore, the molecule might be associated with many alarming health phenomena including premature onset of puberty, feminization in males [15], an increased risk of mammary gland tumors in females [16, 17] and prostate cancer in males [18], infertility [19, 20], hyperglycemia and insulin tolerance [21], and atherosclerosis [22].

In dentistry, BPA is used for the synthesis of Bis-GMA and several other bis-phenyl monomers (i.e. Bis-EMA, Bis-DMA, Bis-MEPΡ), polycarbonate brackets, elastomeric ligatures, and aligners among others. Regarding aligner therapy, it is of great importance to ensure that the aligner material itself and the composite attachments are biocompatible and do not pose a threat to the health of patients. While most investigators failed to find traceable amounts of BPA released from clear aligner materials [23–26] and cytotoxic or estrogenic effects after exposure [27–29], others reported a low level of BPA release from various aligners [30, 31]. Concerning dental resin adhesives, some authors did not detect relevant leaching of hazardous factors or an estrogenic effect [32, 33], while others found considerable BPA release [34], whereby the level peaked shortly after bonding [35–37] and was in correlation to the degree of conversion achieved during light-curing [38]. The bioavailability of the main resin composite monomers, such as BisGMA, TEGDMA, UDMA etc., has been associated with molecular toxicological effects towards the pulp in tooth restorations [39], adverse reactions of gingiva and oral mucosa, and extraoral allergic reactions [40].

Although research has addressed the potential release of factors from aligner materials and dental adhesives independently, to date there is no data on the leaching during aligner treatment in conjunction with resin attachments in a clinical setting. Intraoral conditions and the development of friction and attrition between the sometimes-bulky attachments and the softer aligner material may promote the release of BPA and other compounds. Hence, the aim of the present study was to investigate the elution of substances qualitatively and quantitatively from polyester-urethane aligners and attachments in vivo. The null hypothesis was that there is no identification, at the threshold values, of the analytical instrumentation from aligner- and attachment-derived compounds at any of the timeframes selected.

## Materials and methods

### Patient enrolment

Patients treated with a polyester-urethane aligners (Invisalign™, Align Technology, San Jose, CA, USA) and who were planned to have their attachments removed and who did not have composite resin restorations were enrolled in this study. In the absence of related clinically derived data, the range of compounds (where applicable) identified in vitro were used to calculate a sample size. An ethical approval by the Dental Association of the prefecture of the private practice where the investigation was performed was obtained (No 451/2023). Patients (7 female and 4 male) had a mean age of 24.2 years (standard deviation 11.1; range 14.0–47.0), were in treatment for mean of 20.3 months (range 13.0–33.0 months) and had received an average of 22 attachments (range 15–29) with an average of 16 attachments per patient for the entire period of treatment. All attachments were fabricated with the same flowable light-curing resin composite (Table 1) irradiated for 20 s each, with a LED curing unit emitting 1200 mW/cm^2^ light intensity (Valo, Cordless LED curing light, Ultradent, South Jordan, UT, USA).

**Table 1:** The composition of the flowable composite used for attachment fabrication

Flowable composite	Composition*	Manufacturer
Tetric Evoflow	*Resin*: Bis-GMA, UDMA, DCDMA, TPO, Drometrizol *Fillers*: Ba-glass filler, mixed oxide fillers, YbF_3_, highly dispersed SiO_2_, prepolymers (61.5% wt).	Ivoclar Vivadent, Schaan Liechtenstein

* According to the manufacturer’s information. Bis-GMA: Bisphenol glycidyl dimethacrylate, UDMA: urethane dimethacrylate, DCDMA: 1.10- decanediol dimethacrylate, TPO: diphenyl (2,4,6- trimethylbenzoyl)phosphine oxide, Drometrizol: 2- (2’-hydroxy-5’-methylphenyl) benzotriazole

## Sample size calculation

No sample size calculation based on previous published studies could be undertaken, since this is the first of its kind. Pilot analysis of three initial samples found mean BPA concentration with attachments of 6.5 µg/L with a standard deviation of 2.0 µg/L. Assuming that after removal of the attachment this should be reduced by at least 50%, a common standard deviation, setting alpha at 5% and beta at 20%, 8 samples would be needed at each timepoint. This was increased by 30% to 11 samples per timepoint to account for potential lost samples.

### Collection of rinsing samples

Patients were instructed to rinse with 50 mL of distilled water upon entry and a rinsing solution was collected after this process. Then, attachments were removed with low-speed tungsten carbide burs for adhesive residue removal (REF123-604-30, Dentaurum, Ispringen, Germany) operated at 30,000–40,000 rpm. The patients rinsed thoroughly with water immediately after the grinding process and the sample was discarded, to avoid interference of grinding particles. Subsequently, a second water-rinsing was performed, and the solution intended for analysis was collected. All rinsing solutions, averaging 50 mL, were frozen and coded with the number of patient and time frame without identification of patient’s data.

### Targeted analysis of eluents

Analytical standards of urethane dimethacrylate, UDMA (CAS: 72869-86-4, Purity: 98.9%, Biosynth Carbosynth, Compton, UK), 1,10-decanediol dimethacrylate, DCDMA (CAS: 6701-13-9, Purity: 97.0%, A2S, Saint-Jean-d’Illac, France), bisphenol A glycidyl methacrylate, Bis-GMA (CAS: 1565-94-2, Purity > 97.25%, Sigma Aldrich), BPA (CAS: 80-05-7, > 99%, Sigma-Aldrich, St. Louis, USA) and the isotopic labeled BPA-d16, used as an internal standard, IS (98.1%. Dr. Ehrenstorfer GmbH, Augsburg, Germany) were utilized to prepare stock solutions of each compound (1 mg/mL in methanol) that were stored at − 20 ^o^C.

Acetonitrile and methanol of LC-MS grade (≥ 99.9%) were purchased by Fisher Chemical. Formic acid (98–100%) was supplied by Riedel-de Haen. Ultra-pure water (18.2 MΩ/cm at 25 ^o^C) was produced in the lab using a Temak TSDW10 water purification system (TEMAK, Athens, Greece).

Targeted analysis was carried out using a TSQ Quantum Discovery Max triple-stage quadrupole mass spectrometer with both electron spray ionization (ESI) and atmospheric pressure chemical ionization (APCI) sources, coupled to a Finnigan Surveyor LC system, equipped with a Finnigan Surveyor AS autosampler (Thermo Fisher Scientific, Waltham, MA, USA) and a chromatographic column (Atlantis T3, 2.1 mm x 100 mm, 3 μm, Waters, Wexford, Ireland). Data processing was performed using Xcalibur software 2.1 SP 1160 (Thermo Fisher Scientific).

For the determination of BPA, all the samples were spiked with BPA-d16 (internal standard, IS) at final concentration of 50 µg/L and analyzed by liquid chromatography-tandem mass spectrometry (LC-MS/MS) with APCI negative ionization. Chromatographic separation was achieved with isocratic elution with 50% acetonitrile-50% ultrapure water. For the determination of UDMA, Bis-GMA and DCDMA, samples were analyzed LC-MS/MS with ESI positive ionization. Chromatographic separation was achieved by gradient elution with mobile phase consisting of (a): acetonitrile and (b) ultrapure water both containing 0.1% formic acid and the following program: 20% A (held for 2 min), increase to 65% A in 1 min and to 90% A in 5 min (held for 12 min). Detection of target compounds was carried out in multiple reaction monitoring mode (MRM) using the most intense and characteristic precursor/product ion transitions (Table 2).

**Table 2:** MS/MS detection parameters of UDMA, Bis-GMA, DCDMA, BPA and BPA-d16 (IS)

Compound	t_R_ (min)	Precursor ion (*m/z*)	Product ion (*m/z*)	Collision energy (eV)
BPA	4.0	227.0 [M-H]^−^	93.0	40
			133.1	31
			211.1	34
			212.1^Q^	20
BPA-d16 (IS)	3.9	241.0 [M-D]^−^	223.1^Q^	23
			142.1	28
UDMA	13.0	493.1 [M + Na]^+^	493.1	2
			407.2 ^Q^	26
			321.1	32
			319.1	31
			113.0	34
Bis-GMA	13.5	513.2 [M + H]^+^	513.2	2
			173.0	22
			143.0 ^Q^	20
DCDMA	19.2	311.2 [M + H]^+^	97.1	14
			87.1 ^Q^	17
			83.1 ^Q^	14
			55.1	36

Bis-GMA, bisphenol A-glycidyl methacrylate; BPA, bisphenol A; DCDMA, 1,10-decanediol dimethacrylate; UDMA, urethane dimethacrylate

^Q^ *Quantifier ion(s)*

### Untargeted analysis

Suspect screening and untargeted analysis of the patients’ oral rinse samples was performed with an Orbitrap Q Exactive Plus high-resolution mass spectrometer, HRMS coupled to an Ultimate 3000 series LC pump and autosampler (Thermo Scientific). Mobile phase consisted of (A) 90% acetonitrile-10% ultrapure water and (B) ultrapure water both containing 5 mM ammonium formate and 0.02% formic acid. The gradient program was as follow: 5% A (held for 3 min), increase to 65% A in 3 min (held for 4 min) and to 90% A in 10 min (held for 10 min). Compounds ionization was performed with ESI source in positive mode and data acquisition was carried out in data dependent mode (DDA). Full MS spectra were obtained with a resolution of 70,000 and scan range m/z 70-1050. The four most intense ions of the full scan were fragmented with stepped collision energy of 10, 30 and 50. Data processing for the annotation of the compounds was performed with Compound Discoverer 3.2TM software (Thermo Fisher Scientific, Waltham, MA). A data processing workflow was developed, in which an in-house mass list of 115 compounds correlated to dental resins for suspect screening [41–43] and the mass list of Extractables and Leachables HRAM Compounds provided by Compound Discoverer were incorporated. Additionally, a search on mzCloud [44], a web-based mass spectral database with high- and low-resolution mass spectra acquired under experimental conditions, for untargeted screening was performed as part of Compound Discoverer data processing workflow enabling the compound annotation based on the similarity of fragmentation spectra. All the compounds were tentatively identified without the use of pure standard compounds by using MS data at a confidence level 2 for metabolomics analysis [45].

### Statistical analysis

Normality was assessed through visual inspection of distribution plots and formally with the Shapiro-Wilk test. Descriptive statistics included absolute / relative frequencies for binary variables and medians with Interquartile Ranges (IQR) for continuous variables, as testing indicated skewed distribution. Differences between different timepoints were assessed with Kruskal-Wallis test, two-sided p values, alpha set at 5%, and an openly provided dataset [46].

## Results

**Fig. 1:**
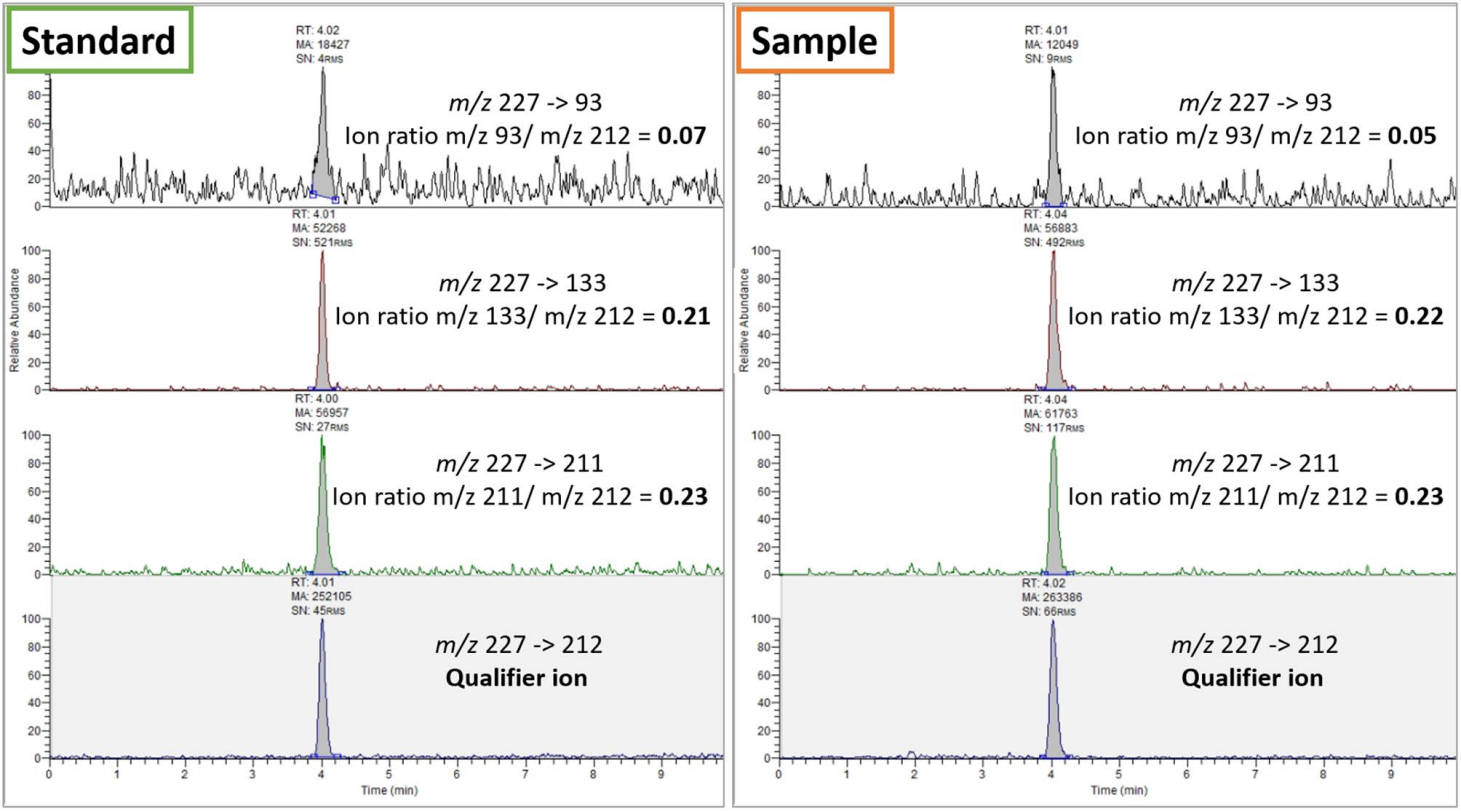
MRM chromatograms obtained from confirmatory analysis of a sample found positive for BPA in comparison to the standard solution at a concentration of 10 µg/L

**Fig. 2:**
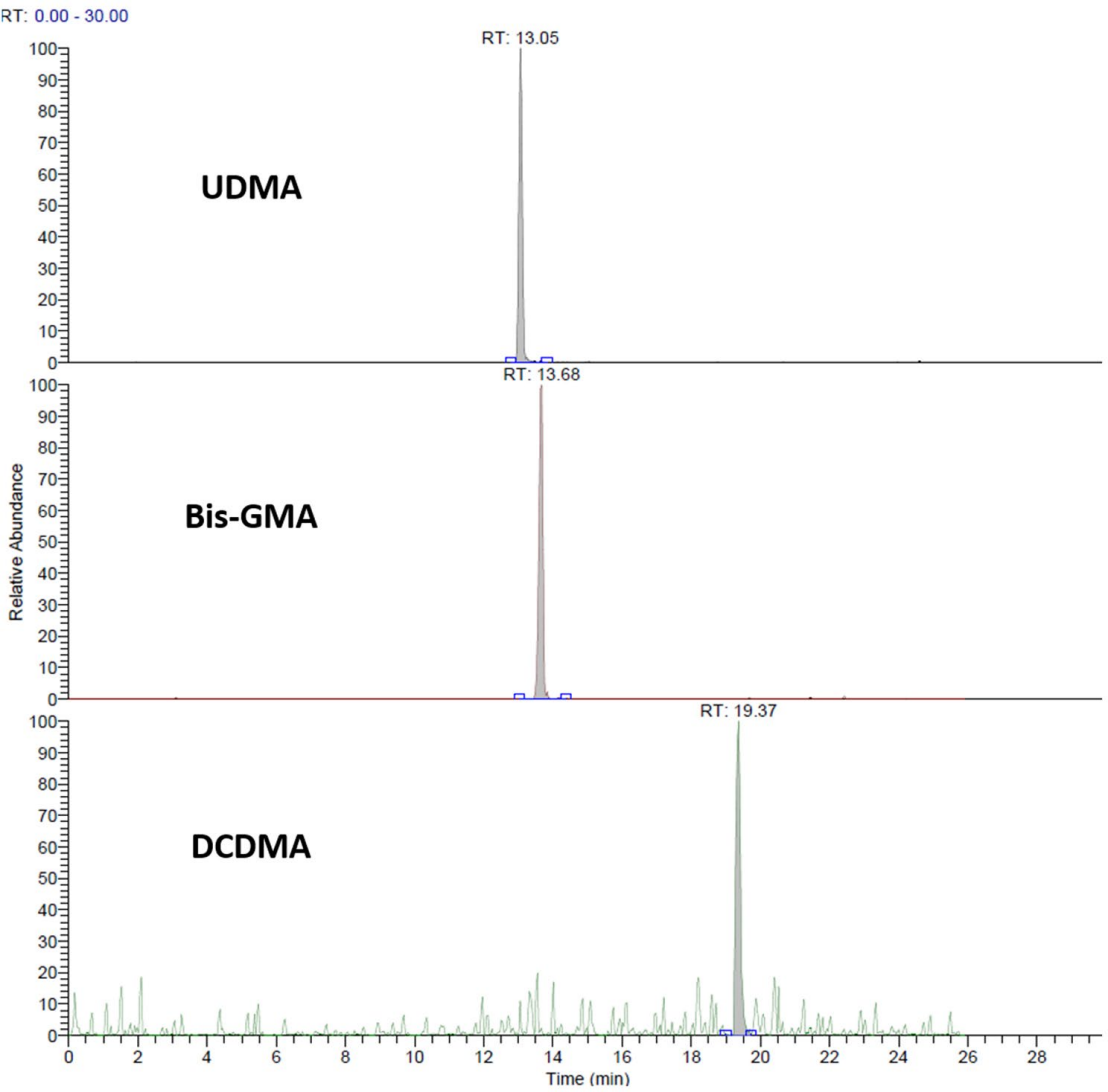
MRM chromatograms obtained by LC-MS/MS analysis of a sample found positive for UDMA, Bis-GMA and DCDMA

In total, samples of 11 patients for 2-time intervals (before and after removal of attachments), each in triplicate, were processed for analysis. MRM chromatograms obtained from confirmatory analysis of a sample found positive for BPA in comparison to the standard solution (10 µg/L BPA) are depicted in Fig. 1. MRM chromatograms obtained from a sample positive to UDMA, Bis-GMA and DCDMA are illustrated in Fig. 2. Identification of the compounds was achieved by comparison with analytical standards.

The results of the targeted analysis are presented in Table 3. The concentration of BPA in the rinsing solution of patients before removal of attachments was four times greater than the corresponding concentration after attachment removal. A large variability of the BPA concentration was observed in some samples, potentially being associated with the size, number, and duration of attachment service intraorally. Of the three targeted monomers (UDMA, Bis-GMA and DCDMA), only UDMA and Bis-GMA were identified at the second timeframe (after removal of the attachments).

**Table 3:** Concentrations (µg/L) of targeted compounds identified in rinsing solutions before and after attachment removal with the corresponding LOD and LOQ values (median values and interquartile range, *n* = 11)

Timepoint	Assessment	BPA	UDMA	Bis-GMA	DCDMA
LOD		0.25	0.25	5	5
LOQ		1	1	15	15
Before attachment removal	Positive	8 / 11	1 / 11	1 / 11	1 / 11
	Quantification	8.4* (4.6–18.4)	< LOD**	< LOD**	< LOD
After attachment removal	Positive	8 / 11	11 / 11	9 / 11	1 / 11
	Quantification	2.7 (1.6–3.9)	4.1 (2.7–4.9)	28.7 (21.3–48.9)	< LOQ
	P_before−after_ (Mann-Whitney)	0.03	-	-	-

Bis-GMA, bisphenol A-glycidyl methacrylate; BPA, bisphenol A; DCDMA, 1,10 - decanediol dimethacrylate; LOD, limit of detection; LOQ, limit of quantification; UDMA, urethane dimethacrylate

*p<0.05 (before - after)  **No analysis was necessary for the compounds which were not detected before attachment removal and were identified after removal

**Fig. 3:**
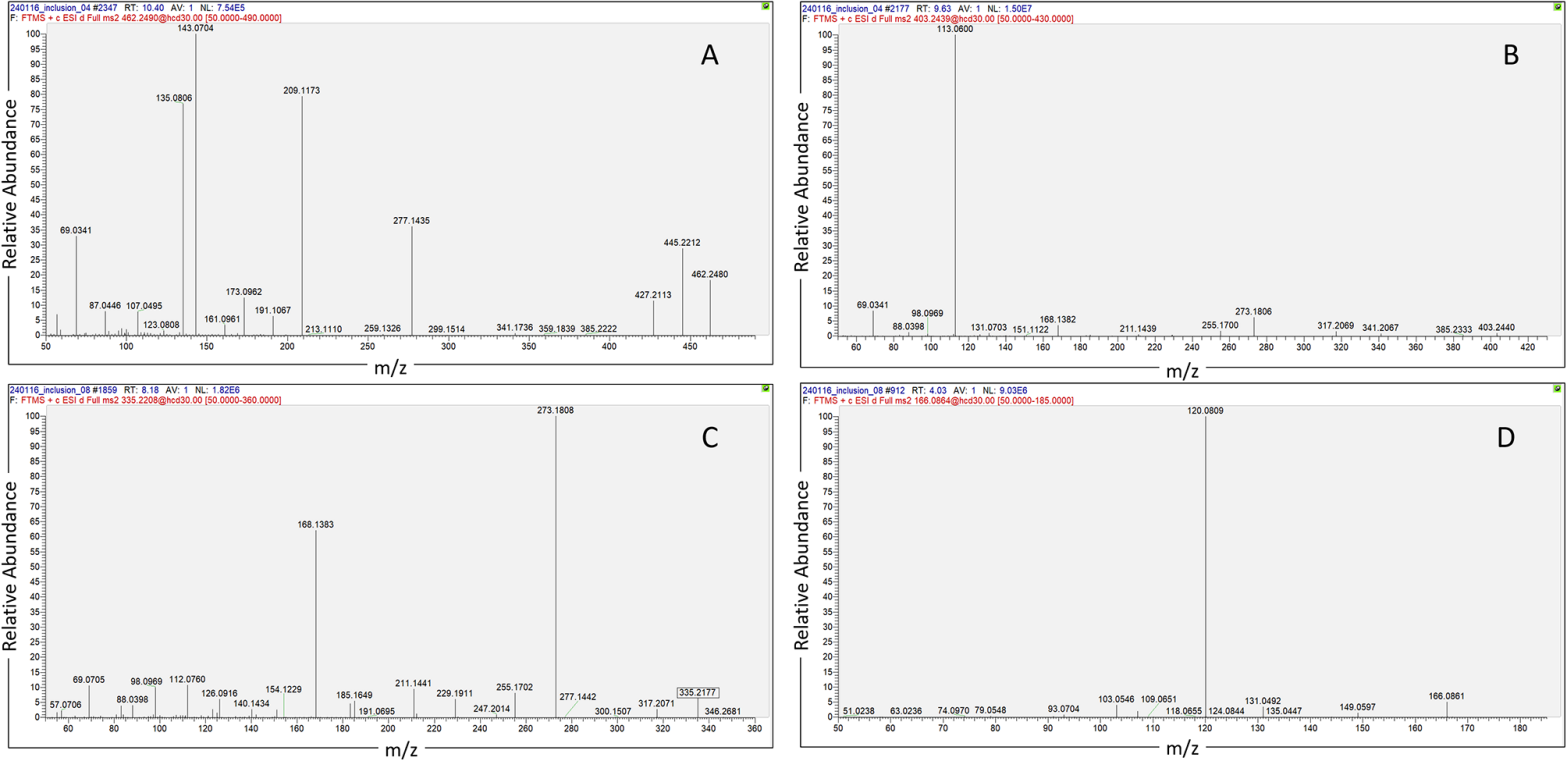
Fragmentation mass spectra of monomer degradation products annotated by untargeted analysis. (**A**) BisGMA-M1 with precursor ion of [M + NH_4_]^+^, *m/z* 462.2490, corresponding to the chemical formula C_25_H_32_O_7_; (**B**) UDMA-M1 with precursor ion of [M + H]^+^,*m/z* 403.2439, corresponding to the chemical formula C_19_H_34_N_2_O_7_; (**C**) UDMA-M2 with precursor ion of [M + H]^+^, *m/z* 335.2208, corresponding to the chemical formula C_15_H_30_N_2_O_6_ and (**D**) DMAB with precursor ion of [M + H]^+^, *m/z* 166.0864, corresponding to the chemical formula C_9_H_11_NO_2_

Table 4 summarizes the results of the untargeted analysis. Mono-methacrylate transformation products of Bis-GMA (Bis-GMA-M1) and UDMA (UDMA-M1), UDMA without the two methacrylate moieties (UDMA-M2), and 4-(dimethylamino) benzoic acid (DMAB), the degradation product of the photo-initiator ethyl-4-(dimethylamino) benzoate (EDMAB), were annotated after attachment removal. Figure 3 demonstrates representative fragmentation spectra of degradation resin products annotated by the untargeted analysis. Several amino acids and endogenous metabolites were also found both before and after removal.

**Table 4:** Results of untargeted analysis of the rinsing solutions before and after attachment removal

Compound	Before removal	After removal
Bis-GMA-M1	-	+
UDMA-M1	-	+
UDMA-M2	-	+
DMAB	-	+
Dibenzylamine	+	+

Amino Acids: Pro, Arg, Gln, Tyr, Ile, Lys, His (before and after)

Endogenous metabolites: Acetylcarnitine, acetylcholine, N-acetyl-D-lactosamine, acetylneuraminic acid, caffeine, creatine, glycylproline, 4-guanidinobutyric acid, hypoxanthine, paraxantine propionylcarnitine, theobromine (before and after)

Extractables/leachables: Dibenzylamine (before and after)

## Discussion

The results demonstrate elevated levels of traceable BPA in patients’ oral rinse samples at the end of clear aligner therapy in conjunction with bonded resin attachments. After the removal of attachments and thorough rinsing, there were still detectable traces of BPA, however, the level was significantly reduced. This may suggest a link between the presence of composite attachments and BPA release. Although rinsing and swallowing during treatment can substantially reduce the initial BPA levels [37], BPA was still detectable even after a mean of 12 months in the oral cavity, implying that the cumulative release during the time elapsed should be much higher. Upon removal of the attachments, the BPA levels showed a marked decline. Contrary to BPA, the attachment monomers were detectable only after removal. Hence, the attachment-derived compounds could be identified in the samples, the null hypothesis must be rejected.

Sampling of rinsing solutions was performed under the same conditions, with patients been exposed to the attachment removal procedure and the first water rinsing, which was discarded to remove interferences from abraded composite particles. The reduction of the BPA content in the solutions after attachment removal by a factor of 3 may imply that the attachments were implicated in BPA release. It seems that before attachment removal the BPA levels were in a low-release equilibrium, which was further reduced after removal. There are two potential sources of BPA; impurities from the Bis-GMA synthesis route or from Bis-GMA degradation of the attachments [47] since the aligner material (polyester-urethane) does not contain bisphenol rings. The prolonged exposure of the attachments in the oral environment and the mechanical fatigue induced by the weekly-changing aligners to the same attachments may enhance the degradation of Bis-GMA, creating a source of BPA.

This may be a gradient release, since the entire attachment, excluding the bonded surface, is exposed intraorally, and subjected among others to repeated installation and removal forces by the aligner. The contribution of the attachment particles produced during attachment removal to the amount of BPA traced should not be excluded, since it has been shown that these particles act as vehicles of BPA and composite monomers [48].

For the main monomers of the resin composite (Bis-GMA, UDMA, DCDMA) no values above the detection limit were registered before attachment removal. This was expected, since high amounts of residual monomers are released in immersion media during the early stages after polymerization, reaching a low-release equilibrium overtime [49]. Nevertheless, after attachment removal, all basic monomers were traced, with Bis-GMA and UDMA being at quantifiable levels. Grinding and inevitably leaving freshly exposed remnants on enamel (i.e., resin tags) may introduce a source for the release of those substances. However, detecting resin monomers in a random, instantaneous sampling immediately after removal of attachments, which were in intraoral service for a year, supports the aspect that particle remnants of the ground adhesives in the oral cavity eluted these monomers rather than release from the exposed resin tags [48].

The presence of monomer degradation products such as Bis-GMA-M1, UDMA-M1 (monomethacrylate) and UDMA-M2 (without methacrylates) was annotated only after removal of attachments. Although such hydrolysates have been traced after aqueous storage [50], detection of these compounds after attachment removal may be assigned to exposure of bulk structure with entrapped degradation products or to compounds produced due to the thermal effects induced during attachment removal by grinding. The presence of dibenzylamine was also detected in patient’s oral rinses samples both before and after removal. Dibenzylamine is a secondary amine commonly used in the production of certain polymers, in particular as constituent of polyurethane and polyurea compositions having excellent processability and high flexibility, and also constituent of epoxy resin compositions [51]. A recent *in silico* study revealed the possible hepatotoxicity of dibenzylamine as well as its high affinity with several cytokines [52].

So far, the evidence for possible leaching of harmful substances and the concomitant risk of adverse health implications from aligner therapy remain vague and ambiguous. Contrary to the presented results, in vitro investigations have failed to show leaching of BPA or other monomers [23–26] and induction of cytotoxic or estrogenic effects [27–29], mainly because of the model used which does not include attachments or does so in limited extent and not under in the multiplicity of the factors present in the actual clinical environmental milieu. Intraorally, temperature and acidity fluctuations, masticatory loads, attrition of the aligner and/or resin during fitting of an active aligner, presence of microbial metabolites or enzymes with a degradation capacity to esters among others, may modify the type, kinetics and amount of compounds released. Studies assessing as-received [23, 24, 27, 28] and retrieved [25, 26, 29] aligners report similar results, although ageing in the oral environment substantially affects the integrity of the material. Whereas aligners are usually changed every two weeks, the mechanical properties are adversely altered even after one week of use compared to new aligners. The Marten’s hardness and indentation modulus of the material are reduced while it displays an increased elastic index [53, 54]. The reduction in hardness and roughness is indicative of a higher susceptibility to wear, suggesting that aged aligners may be more prone to the release of harmful factors. However, previous research does not support this assumption, as no leaching was detected even after intraoral or in-vitro ageing [25, 26, 29], which complies with the lack of changes in the chemical composition between new and used aligners [53], when these are aged in vitro and without the use of attachments.

In contrast to these findings, the results of the presented research are in line with a recent laboratory study [30] and a single randomized clinical trial [31], which report traceable amounts of BPA leaching. In the only in vivo study up to date, patients who received vacuum-formed retainers (Essix ACE Plastic) showed increased salivary BPA levels even after one month of retainer wear, with a peak after one week [31]. Although the investigation was also conducted in a clinical setting and the results substantiate the current findings, a comparison has to be done cautiously as another aligner material with different compositions and mechanical characteristics [54] was analyzed. In general, the difference in sample composition, preparation and storage makes a standardized comparison between studies difficult.

All of the aforementioned studies assessed the potential leaching of the aligner material itself and neglected the possible release of molecules from resin-based attachments, which nowadays are most often used as auxiliaries in aligner therapy. To date, no study has directly addressed the possible release of harmful factors from attachments used in aligner therapy, specifically BPA, which derives from the degradation of attachment resins [34–36, 50]. One clinical trial found elevated levels of BPA after bonding orthodontic brackets and a return to baseline levels after thorough rinsing [37]. However, the leaching from the bulky attachments used in conjunction with aligners presumably differs from the adhesive applied in a sandwich-like pattern during bracket bonding. First, attachments have a higher surface-area-to-volume ratio, with the exposed surface area of the adhesive estimated to be many times higher than with conventional brackets, where only the margins of the adhesive are exposed [8]. Therefore, the potential reactivity of the attachments with the surrounding oral environment is multiplied. Second, the attachments are exposed to daily placement and removal of the aligners and to masticatory stress, which may lead to abrasion and attachment surface alterations. According to previous research, the attachment’s texture noticeably changes over a treatment period of six months, displaying cracks and fractures. Also, the attachment wear is influenced by the filler loading of the resin, whereby a micro-filled composite displayed greater wear than a nano-filled composite [53]. In addition, composite resin and human enamel have a 6-fold and 23-fold increased hardness compared to Invisalign™ aligners respectively [54]. It could therefore be anticipated that aligners are more prone to wear when in contact with enamel, however, the surface morphology and roughness may be more decisive in this case. Consequently, attachments may not only release factors but also influence the leaching of the aligner material in service.

Although the presented data suggests the relevance of attachments in the release of potentially harmful molecules during aligner treatment, their impact cannot conclusively be confirmed. The detected traces of BPA in this study compared to previous research, where no leaching of aligner materials was found [23–26], indicate the potential reactivity of the attachments with the oral environment. On the other hand, the results of a recent clinical study reporting BPA traces after thermoplastic retainer wear [31] are not in line with this assumption and urge the relevance of the aligners’ behavior in a clinical setting compared to strict experimental conditions, suggesting that aligners, when used without attachments may show no release of BPA and resinous degradation products.

Considering the feasible estrogenicity and cytotoxicity of the released factors and the limited evidence on the subject so far, appropriate risk management should be applied by practitioners and the number of attachments should be reduced to the absolute necessary, including alternative materials for attachments, such as BPA-free resins or BPA-free resin bonded buttons of medical grade polymers (i.e., ultrahigh molecular weight polyethylene) with improved biological properties. Larger scale clinical investigations should explore the intraoral release of compounds in patients treated with Invisalign and 3D-printed aligners to assess the potentially hazardous elution of biologically-active species along with the release of microplastics.

## Conclusion

Elevated levels of BPA and of resin monomers Bis-GMA and UDMA as well as the presence of degradation monomer compounds (Bis-GMA-M1, UDMA-M1 and UDMA-M2) were traced instantaneously in patients treated with Invisalign™ and flowable resin composite attachments for 20 months period, implying that the cumulative release may be higher than the amounts reported in this study. Alternative resin formulations and attachment materials may be utilized to reduce eluents.

## Data Availability

The study’s dataset is openly available through Zenodo (10.5281/zenodo.10926518).
